# Bibliographical Notices

**Published:** 1837-07-01

**Authors:** 


### Periscope;

OR,

### CIRCUMSPECTIVE REVIEW.

"Ore trahit quodcunque potest, atque addit acervo."

### Notices of some New Works.

The British Flora Medica; or, History of the Medicinal Plants of Great Britain. Illustrated by a Coloured Figure of each Plant. By Benjamin H. Barton, F.L.S. and Thomas Castle, M.D. F.L.S. Vol. 1. 8vo. pp. 408; and 101 Coloured Figures. London; E. Cox, St. Thomas's-street, Southwark.

We approach this work with a two-fold pleasure. The first is on our own account; the second on that of our readers, and the profession in general. For ourselves, it is delightfully refreshing, after toiling through many an arid waste of letter-press, to enter a flower-garden, where plants of every form and hue, are blooming in all their native loveliness. Through all the dreariness of a tedious winter, we have been presented with a never-failing succession of these elegant creations; which, beautifully coloured as they are, have turned our study into a conservatory.

With respect to our readers, and the medical profession at large, the pleasure which this work affords us, arises from the assured prospect we have of its extensive utility. This utility will extend to various classes of which the profession is made up. To the student, in the first place, it will be invaluable. Without quoting again what has been quoted so often, the classical allusion to the deeper impression made by objects presented to the eye than by those described to the ear, we may lay it down in plain English as a principle, which comes home to every one's experience with all the force of an axiom, that a great saving of time and labour is effected, and a much more accurate and intimate knowledge is acquired, when objects and images themselves, instead of mere verbal descriptions of them, are presented to the senses. Hence the great advantage of specimens, preparations, experiments, and diagrams; and the eminent success of what we may call "the illustrative method," which of late years has been acted on so largely.

This holds good in every science which admits of optical illustration; but botany is a science which pre-eminently demands it. Notwithstanding its minute and accurate language, the most laboured description of a plant will give a less adequate notion of it, than a single glance at the plant itself. But plants are not to be obtained at all times, and in all places. In the heart of a city and the depth of winter, they could not be procured; and even in the country, and with a favorable season, the student is often unable to devote the time necessary for seeking and gathering them. In such circumstances, it is fortunate that a very good substitute for the plants themselves, may be found in representations of them, provided those representations are coloured in strict accordance with nature; and we have no hesitation in saying, that an attentive study of the admirably coloured plates in the work before us, together with a careful perusal of the lucid and interesting details which accompany them, will leave little to be learned by an actual inspection of the plants thus described and illustrated.

To those, again, who are actively engaged in practice, this work will be found of essential service. Occupied, during the day, in a round of professional visits; and paying supreme and almost exclusive attention to the practical details of their art, their theoretical and general knowledge is apt to slide into forgetfulness; unless means are taken occasionally to revive and refresh it. One of the most effectual of these means, in the science of Botany, is an illustrated work, which, by the fidelity of its representations, will supply the place of the actual specimens, which other avocations will not permit of being collected. This is just the desideratum which the work before us is calculated to supply. By the inspection of an hour or two in the evening, it will revive fading impressions; and make "the burying-places of the memory give up their dead."

There is yet another class by whom this work will be hailed with peculiar satisfaction. We allude to those who are called to practice in a foreign land; and who, in the midst of gaudy exotics, are in danger of forgetting the humbler plants of home-growth which they have left behind. To these the British Flora Medica, will come with a peculiar charm; and we are persuaded that after the particular and impartial description we shall give of it, no medical man will leave his native shores, without making it an essential article of his outfit.

We shall allow the authors (in the following extract from their preface) to give their own account of the object they had in view, in the publication of this work, an object which (so far as they have yet proceeded) we must candidly acknowledge has been attained to our entire satisfaction.

"The immediate design of this work, is to furnish an accurate description of all the medicinal plants indigenous to Britain, which appear entitled to that character, from the estimation in which they were held by the greatest and most skilful of the old physicians. That they were generally correct in their judgment is apparent from the fact, that several of the plants which they recommended, after having been allowed to sink into oblivion, and to remain there neglected for many years, have again been successfully used by modern practitioners. This is well exemplified in the history of foxglove; the more prominent effects of which, were fully understood in the sixteenth century. It was admitted into the London Pharmacopœia in 1725; was discarded in 1746; and has lately been restored with more than its pristine honours. How many more blest secrets of the earth remain yet to be discovered, it is impossible to predict; but if these pages should succeed in calling attention to one such plant, the labours of the authors will be abundantly recompensed."

In pursuance of the object thus stated, the plan adopted is to give a coloured engraving of every British plant, employed either in professional or domestic medicine; each figure being accompanied by a letter-press description, which is full without being wearisome, and concise without being meagre. The order in which the plants are described, is not systematic, but alphabetical; nor does the latter conform to their scientific names, but to their common appellations; although the former stands first. This arrangement we should have objected to, in a scientific work with a Latin title, were it not that at the end of the volume a table of contents is given, in which the plants are arranged alphabetically according to their scientific names. We presume that at the end of the work an English Index will be given.

The description of each plant comprises its scientific and common name; its class and order in the Linnean arrangement; its place in the natural system; its generic and specific characters; and a remarkably copious list of synonymes. We have been particularly struck with this feature of the work; which comprehends the name of each plant in from ten to fifteen different languages; comprising the Latin and Greek always; sometimes the

Hebrew; most of the European, and many of the Asiatic tongues. After, follows an account of its general appearance and history; its qualities, and medical uses. We give a specimen.

"No. 83. Fumaria Officinalis. *Common Fumitory*. Class 17; *Diadelphia*. Order 1; *Hexandria*. Natural Order; *Fumariaceæ*. Generic Characters. *Calyx* of two deciduous leaves. *Petals* four, irregular, one of them spurred at the base. *Silicle* indehiscent, valveless, one-seeded. *Style* deciduous. Specific Characters. *Silicles* globose, refuse. *Pedicles* fruit-bearing, erect, more than twice as long as the bracteæ. *Leaves* supra-decompound. Leaflets wedge-shaped, cut, with linear-oblong lobes. Synonymes. *Greek; καπνος,* (Dioscorides). *Latin:* Fumaria officinarum, et Dioscoridis (Bauh. Pin. 143). Fumaria purpurea (Ger. Em. 1088). Fumaria vulgaris (Raii Syn. 204. Park. Theatr. 287). Capnos fumaria (Lob. obs. 437). Fumaria officinalis (Lin. Sp. Pl. 984. Eng. Fl. III. p. 255. Eng. Bot. t. 589) *French;* Fumeterre; Fumeterre officinale. *Italian;* Fumosterno; Fumaria. *Spanish;* Fumaria; Palomilla; Hiel deterra. *Portuguese;* Fumaria. *German;* Erdrauch; Gemeiner Erdrauch; Taubenkropf. *Dutch;* Duive-Kervel; Aard-rook; Grysecom. *Danish*; Jordrog; Aakersissel. *Swedish*; Jordrœk. *Polish*; Rutha ptasza; *Persian;* Schahtra. *Japanese;* Fingosakf. *Arabic;* Bucklutulmeric."

As a relief to these necessarily dry details, and as a specimen of the interesting style in which the great body of the work is composed, we quote the general description of the Bellis Perennis, or Common Daisy.

"This modest, crimson-tipped flower, the delight of all ages, is as frequent as it is beautiful. It gems our meadows and pastures from March to November; most abundantly in the height of Spring and the beginning of Summer; but a few may be seen at all seasons. Hence Montgomery says—

'It smiles upon the lap of May;

To sultry August spreads its charms;

Meets cold October on his way;

And twines December's arms.'

And Shelley, in his exquisite dream of Spring-flowers, introduces—

'Daisies, those pearl'd Arcturi of the earth;
The constellated flower that never sets.'

The generic name is derived from the Latin, *bellus* (pretty). It was also called *Margarita* (a pearl); of which there are synonymes in many of the Continental languages. The bold and cheerful manner in which its petals expand to the light of day, and their habit of closing again at night, and against rain, suggested the name of *daisy*, or as Jonson calls it, *day's-eye*. Chaucer, whose affection for this flower manifests itself in many parts of his poems, gave it the same designation in 'Aprilis and his pleasant Showres', and in the 'Legende of goode Women.'

'That well by reason men it call may
The *daisie*, or else the *eye* of the *daie*.'"

We pass on to its "Medical Properties and Uses," which the necessity of economising our space obliges us to condense, as we have done the preceding extract.

"Whatever *poetical* fame may have encircled the daisy's coronet, its reputation as a medicine has dwindled almost to nothing. The ancient physicians were very lavish in its praise: and for its supposed vulnerary properties, it received the pharmacopœial name of '*consolida minima*.' Fabricius and Ettmuller speak of its efficacy in promoting the absorption of extravasated blood. Matthiolus affirms that even penetrating wounds of the thorax have been cured by this remedy; and Konig goes so far as to assert, that in lesion of the lungs themselves, it has proved extremely beneficial. Hence it came to be considered as an anti-pleuritic, and was prescribed in nearly all the diseases to which the lungs are incident, as well as in liver complaints, agues, dropsy, asthma, and scrofulous swellings. Some of the moderns have esteemed it beneficial in phthisical complaints; and it still occupies a place in a few of the Continental pharmacopœias. The tincture of the flowers (in the Wirtemberg pharmacopœia) contains a portion of sulphuric acid; to which its reputed soothing and refrigerent properties are doubtless attributable. The expressed juice of the plant, in the dose of half an ounce, is sometimes given by the poor to their infants as a laxative. They also consider it a remedy for fluor albus. The root of this plant, which is the part most potent in sensible qualities, may probably be found, at some future day, not inert as a medicine."

The preceding extracts are sufficient evidence of a fact, which every page of the work abundantly confirms,—the research and labour employed in its construction. Not only our own favourite English poets, but classic authors of every age, are laid under contribution. Not a single allusion to a plant is allowed to pass without notice and illustration; and the work might be consulted with advantage, in any future edition of our standard Greek and Latin authors. Take the following elegant little *morçeau* from the description of the Acanthus Mollis; which had the honour of having given rise to the Corinthian capital.

"The generic name (derived from *ακανθα, a spine*) does not apply to this species, which is smooth and unarmed. Nevertheless it is *the* Acanthus, *par excellence;*—the plant celebrated by Virgil, Ovid, and other poets. It has been renowned for ages, on account of the beauty of its leaves, which furnished the ancient sculptors and architects with one of their chief ornaments. The Greeks and Romans carved them upon their vases, and their massive goblets; and wove them into their costly vestments. The discovery of their ornamental character is contained in the following legend. A young lady of Corinth having died a few days before her marriage was to have been celebrated, her afflicted nurse put into a basket different articles of which the girl had been fond; and placing it near her tomb, upon a plant of the acanthus, covered it with a large tile. The following spring the acanthus grew up; and its large leaves encompassed the basket; but meeting with the projecting tile, they were curved at the extremity, and bent down. An architect named Callimachus passing by, was struck by the novelty and beauty of the figure, and resolved to apply it to the decoration of the Corinthian capital."

Historical details are introduced, whenever the subject demands or admits it; and have the effect at once of enlivening and enriching the description. We select, as an example, the introduction and early history of the Hop (Humulus Lupulus); the name of which is derived from the Anglo-Saxon *hoppan*, to *climb*. Under the head of this plant, interesting particulars are given of its culture, growth, gathering, preparation for the market, and uses, both general and medical; the latter comprising an investigation of its active principle, lupulin.

"The first mention of hops occurs in a letter of donation by King Pepin, which speaks of humuloraiæ, meaning probably, hop-gardens. Beckmann does not find the word *lupulus* to occur earlier than the eleventh century. About the beginning of the following century, hops were introduced into the breweries of the Netherlands. They seem to have been unknown in England, till brought from Artois about the year 1524. Ale, the common drink of our Saxon ancestors, was made generally from malt alone, or clarified with ground-ivy (hence called *alehoof*) which was thought to render the liquor more wholesome. Parkinson says—'The ale which our forefathers were accustomed only to drink, being a kind of thicker drink than beer, is now almost quite left off to be made; the use of hoppes to be put therein, altering the quality thereof to be much more healthfull.' And Gerard makes a distinction between ale and beer:—'The manifold vertues of hops do manifestly argue the wholesomeness of beer above ale; for the hops rather make it a physicall drinke to keepe the body in health, than an ordinary drinke for the quenching of our thirst.' For several years after the introduction of hops, there was a strong popular prejudice against them. Henry VIII. issued an injunction to the brewers, not to put any hops or brimstone into the ale. Walter Blith, in his 'Improver Improved,' published in 1649, informs us—'That not many years since, the famous city of London petitioned the Parliament against two nuisances; and these were Newcastle coals, in regard of their stench, &c., and hops, in regard they would *spoyle the taste of drink*, and endanger the people.' Now, however, severe penalties are inflicted on brewers who use any other bitters for preserving their beer."

On the subject of poisonous plants, the directions of our authors are very precise. The appearance and characters of such plants, are described with great attention and accuracy; so as to prevent, as far as possible, their being eaten by mistake; and when such an event has unfortunately taken place, the succinct account which is given of the treatment proper to be pursued, will be found of great service in all cases; but especially so in country places, where medical assistance is not immediately at hand. The whole is rendered more impressive, by the relation of a number of interesting cases. We select one or two, under the head of Aconite.

"Willis relates an instance of a man, who accidentally ate some of the leaves in a salad, and died mad in a very short time. Matthiolus mentions the circumstance of four robbers, under sentence of death, to whom this plant was administered; two of whom, after suffering the most violent torments, were saved by appropriate remedies. The other two died. One of the latter a few hours after taking the poison, became idiotic; the face was bathed in a cold sweat; and a total loss of sensation, with fainting and spasms, followed. He vomited a quantity of bilious matter; the body swelled up; and he died in a state of apoplexy.

"In the Philosophical Transactions is related the case of John Crumpler; who, at eight o'clock in the evening, ate a salad into which, by some negligence, a few leaves of the aconite had been introduced. He instantly felt a burning heat in the tongue and gums, and a considerable irritation about the cheeks. He imagined that the blood no longer circulated through the limbs; but felt no disposition to vomit. Perceiving that the symptoms increased, he drank about a pint of oil, and a great quantity of tea, which produced vomiting. The symptoms, however, instead of disappearing, grew worse; and at ten o'clock a surgeon was called in. He was treated with sal volatile, an infusion of the blessed thistle, &c. The next morning he was much better; and soon recovered.

"A family near Lille were poisoned by this plant, in consequence of a tincture of the roots being mistaken for that of a species of lovage. The usual symptoms soon appeared, with swelling of the face, vomiting, and purging. Two of the individuals died. Linnæus relates that an ignorant surgeon, who prescribed the leaves of the aconite, on his patient's refusing to take them, took them himself, and died.

"The writer has lately been informed, on credible authority, of a case of idiotcy supposed to have been occasioned by this plant. The unfortunate sufferer was a young man, much attached to floricultural pursuits. He was suddenly taken ill, with the most violent symptoms produced by that class of poisons to which the aconite belongs; but his maniacal state precluded any satisfactory conjecture as to the precise cause. A celebrated physician was called in, and by vigorous measures, subdued the violence of the disorder; but the mental faculties were fearfully impaired. He had no recollection of his former pursuits; was unable to comprehend the simplest matters; and was, in fact, taught his alphabet a second time. Being obliged to be treated as a mere child, he was one day shewn, for his amusement, a collection of plates, among which there happened to be a figure of the aconite. He immediately exhibited great agitation and alarm, pointing at the plate, and then to his mouth, and uttering incoherent noises. His friends then suspected the true cause of his malady, and concluded that it must have arisen from his having incautiously swallowed some part of the plant, while engaged in his favourite avocation."

The manner in which this work is got up, does the publisher great credit.—The print is clear; and the paper excellent. It is issued in monthly numbers, each of which contains forty-eight pages of letter-press, and twelve coloured figures. Four of the latter are grouped, without being crowded, on a page of thick drawing-paper; and this enables the proprietors to bring each number within the very moderate charge of half-a-crown. The whole will be completed in sixteen numbers; eight of which, forming a remarkably handsome octavo volume, have already been issued.

---

MR. HEADLAND'S ORATION BEFORE THE LONDON MEDICAL SOCIETY. 1837.

Mr. Headland is one of the honorary secretaries, and one of the most distinguished members of this parent institution. He is an observant practitioner, of good education, and excellent natural abilities. We had always looked upon him as strongly inclined to medical aristocracy, and as setting his face against all innovations on our ancient and venerable institutions.—But we find we were much deceived. We ourselves have been often bantered in our projects of "*levelling* upwards" the several grades and denominations of the profession, but Mr. Headland is a *leveller* in the wholesale—or, as some of our contempories will say, no doubt, a leveller downwards.

"The present division of the profession into grades, and the subdivisions in the separate colleges or corporations, you are all so familiar with, that it would be a matter of unnecessary detail to describe them; I would only remind you, that there is scarcely one of *you* whom I have now the pleasure to address who is not by their bye laws excluded from the honours and emoluments of the college or corporation to which he may be attached. Now, I would ask, would not any person unacquainted with the fact, conclude that there must be an essential difference in the education and consequent competency of the several practitioners in medicine, according to their academical rank? would they not be surprised at being told that so far as medical education is concerned, there is *no* essential difference between the Fellow of the College of Physicians and the extra Licentiate of that body; or between the President of the College of Surgeons, and the ordinary member of that body; or between either the physician or the surgeon and the apothecary; but that if strict examination be a test of competency, it is more perfect as regards the lowest of these grades than the highest—nay, that the apothecary is required to give evidence of greater knowledge of the sciences collateral to medicine, that even the physician."

There is much truth, but some fallacy in the above passage. The *medical* education of the three classes is *not* precisely the same; inasmuch as one of the corporate bodies prescribes five years apprenticeship behind the counter, as a component part of the medical education, while the other two bodies leaves the individual to pursue his classical studies where he likes (the Fellows of the College of Physicians at Oxford or Cambridge), and, at a certain age to undergo examination. We admit this tentamen is more searching at Apothecaries' Hall, than in Pall Mall East, or Lincoln's-inn-fields; but the apprenticeship renders the examination more onerous, on account of the number of years sacrificed in dispensing medicine.

The following complaint is put in strong language, and we think the cause of the complaint is a little exaggerated.

"Yet, after the many toils he has endured to obtain this proof, he who succeeds is contemptuously termed an *apothecary, from my lord to the shop boy*, and deemed subordinate to all other ranks in his profession—and why? Is it because he has no knowledge of medicine and the sciences that belong thereto—or because he has not the preliminary of a good education? No, for his examination is a proof of the sufficiency of both; but because he belongs to what by custom is considered the lowest grade of his profession.—And so it is with the profession itself—each in the ascending scale endeavours to elevate himself upon the supposed inferiority of the one below him, from the Fellow of the College to the Licentiate of the Apothecaries' Company."

We apprehend, from some observation and experience, that the public entertain no such *contempt* for general practitioners as our author infers—and that "from the lord to the shop-boy," the public know very little of the education or qualifications of the different denominations of the profession. This, however, they know and act upon.—They very probably conceive that there is no difference in education or examination; but they see that one man takes to physic, another to surgery, a third to ophthalmic, a fourth to dental practice—and a fifth to all combined. It is not very unnatural for the public to conclude, on the principle of division of labour, that he who restricts himself to any one branch (allowing that the original education was equal), may acquire greater experience and skill in that branch than those who cultivate another, or all the other branches of the art. This, we think, is both natural and just. And hence it is, that, in dangerous cases of physic, the physician's assistance will be called in—and that without any insult to the surgeon or general practitioner—and so will the pure surgeon (as he is called) in dangerous cases of surgery. Neither do we find the general practitioner either offended or vexed by these consultations. There are difficulties enough in our practice, and serious responsibilities attached to our decisions, to make us all very glad occasionally to have the co-operation of our brethren—and we ourselves have met Mr. Headland in consultation where there was anything but bad feeling on either side. But enough of this.

Mr. Headland has descanted with energy and eloquence on the beneficial influence and effects of medical societies. We can confirm his observations on this point, for we can conscientiously affirm that we never left a meeting of this description without bringing away with us some additions to our practical knowledge that were afterwards available at the bed-side of sickness.

---

A Treatise on the Prevention and Cure of Pulmonary Consumption. By Robert Little, M.D. 8vo. pp. 160. 1836.

This is a respectable compilation in a small form, and calculated to do some good amongst non-professional readers—especially the second chapter, on the prevention of pulmonary consumption,—but surely Dr. Little must have greatly deceived himself if he thought that the fine thread with which he sewed together the facts and observations of others, could be taken for the substantial tissues which it connected. It is very true that the author has interwoven slight commentaries with the investigations of his predecessors, but very few will take the trouble of searching these out from the mass of well-known matters in which they are overwhelmed. He thinks, indeed, that if his principles and practice of *hygiene* were carried into effect, there would be a "*vast reduction in the number of victims*" to pulmonary consumption. Of this we have some doubt. But of the impracticability of carrying his hygienic plans into execution, upon any scale that would produce even a very trifling *reduction* of the malady we have no doubt at all. Thus, the preventive check is applied to the *fœtus in utero*, which is certainly taking time by the forelock! "During the whole period of utero-gestation, the system of the mother is to be kept in a vigorous state." How is this to be done amongst the poor, where the disease is so prevalent? And we answer for it that the means taken to keep the wives of the rich in the greatest state of vigour, would be just those which would tend to increase abortion and various diseases—namely, good eating and drinking. Dr. Little, however, does not give us any rules for this invigoration of the pregnant female, but passes on to the *post partum* state of the child, telling us that *no female*, of a scrofulous or consumptive habit, should be allowed to suckle her child. This is another specimen of the visionary and impracticable schemes of our author. But even were it possible to carry the rule into effect in this country, where nearly half the mothers and nurses would come under the proscription, how rapidly would the population be increased, beyond its already redundant condition!

While, however, the hygienic precepts of Dr. Little are inapplicable to the masses of the population, and consequently inoperative in the reduction of phthisical ravages in this country, we do not deny that his suggestions may be usefully put in execution in the cases of a few solitary individuals happily circumstanced, and philosophically constituted. We will quote the doctor's own words in proof of these assertions.

"With reference to the infants of those who enjoy all the comforts of life, it is very easy to carry into effect any directions which are laid down respecting food, air, exercise, clothing, or any other thing which is requisite, at that stage of life, for the purpose of giving tone to the system; but the same is not the case with the poor, who, in ninety-nine cases out of a hundred, cannot possibly provide for their infants almost any one thing that would be suitable for their tender age."

Instead of 99 in the hundred who are precluded from Dr. Little's hygiene by the *res angusta domi*, he might have said 999 out of 1,000! With these reflections we part from Dr. Little's work, reiterating our opinion, that it can be serviceable to only a very few, and that in it the author has evinced assiduity and research that might be more advantageously employed in some other direction.

---

### PHILOSOPHY OF LIVING.

1. THE PHILOSOPHY OF LIVING. By —— TICKNOR, M.D. New York, 2d Ed. 1836.
2. THE PHILOSOPHY OF LIVING. By HERBERT MAYO, F.R.S. London, 1837.

It is not a little curious that two authors divided from each other by the vast Atlantic, should have stumbled upon the very same title for their respective works! It is true that there was time enough for the British writer to have seen the transatlantic volume before he published his own, since the American work reached a second edition, and is reprinted in the American family library, of 1836. Still we do not suspect the least piracy in the case—for though the two works have the same title, and are nearly on the same plan, yet the execution of the one is totally different from the other. They both go into minute details on the diversities of constitution, diet, digestion, exercise, sleep, bathing, clothing, air, passions, education, &c. &c. They thus traverse roads that have been beaten as smooth as the blocks of stone in the Via Sacra, when Rome poured forth her millions of inhabitants daily. That there is a good deal of original matter interwoven with established and well-known materials, in these volumes, we readily grant; but, from the hacknied nature of the subjects, none but creative geniuses could impart much novelty now to the "Philosophy of Living." Of the transatlantic author we must hint our suspicion that he has much enthusiasm in his constitution, and consequently that some of his portraits of civilization, fashion, and refinement in the United States are "un peu trop fort." The extract which we have taken from his book on the "Snuff-chewing" of the fair Americans will bear us out in this remark. The English author is very circumspect in his observations, though he appears to have had a good deal of difficulty in steering between two dangers—that of entering too deeply into physiology for the general reader, on the one hand—or of being accounted superficial by the professional critic. The former of the two he is most anxious to avoid, and the following reason will be considered rather curious—though we think it by no means a bad one.

"An author on *Hygiène* must, therefore, be content to skim the surface of knowledge, if he desires his work to be read by those to whom it can convey information. The cream, however, is often on the surface: and I believe it to be generally true, that superficial books are the most useful. Such is my defence, if, in the attempt to suit this volume to unprofessional readers, I have rendered it too trivial and discursive. I may be permitted to add, that I should not have ventured upon a treatise of so popular a character, had I not already given evidence, in former publications, of having laboured at severer professional studies."

No one will suspect Mr. Mayo of inability to write minutely on all points connected with anatomy, physiology, pathology, or surgery; but there may be some who think that a young hospital surgeon can scarcely have had such amplitude of medical practice as to authorize a volume or volumes on matters peculiarly connected with the experience of the physician. We see proofs of this position in various parts of the work where the *lamp* is more perceptible than the bed-chamber of the sick. We may refer to the section on "Bathing," for illustration, and which, among some speculative opinions, the following passage will be found.

"It is believed that there exists a connexion between gout and acidity, difficult as it is to trace the link, or to follow its influence through the various stages of assimilation. Now the skin is a means of escape for superabundant acid. The perspiration is acid, and in proportion as it is profuse, it more completely rids the system of this principle. Theoretically, therefore, the vapour-bath should be a preventive of gout; and in practice I have seen its good effects; and have known great benefit derived from it, in cases where the diathesis has been strong, and no contravening circumstances have been present."

We apprehend that the beneficial effects of vapour-baths in gout, might be accounted for on other principles than the elimination of acid from the constitution. However, we have found little to criticise in Mr. Mayo's work, and much to approve. Although professedly written for the benefit of the general reader, we can assure the medical peruser of this volume, that he will find much to amuse and instruct him. We shall introduce some extracts from the work in another part of the Journal—and the same of Dr. Tickner's publication.

---

THE WORKS OF JOHN HUNTER, F.R.S. WITH NOTES. Edited by JAMES F. PALMER, Senior Surgeon to the St. George's and St. James's Dispensary, &c. &c. In Four Volumes, illustrated by a Volume of Plates, in Quarto. Vols. I. and II. 8vo. pp. 632—488—London, 1835.

*Fasciculus of Plates to the Two Volumes.*

John Hunter has become so national an object of respect and pride, that a complete and a splendid edition of his works may be said to be absolutely necessary. There is no author with whom English surgeons should be more familiar, for our present ideas and practice are in a very great degree derived from him; yet, singularly enough, there is no author who has been generally less read and more robbed than he has been.

Much of the neglect which has been extended to his writings has arisen from the obscurity and involved structure of his language—more from the circumstance of his having been the pioneer of the modern improvements, and from those who have extended his researches having freely made use of his facts and principles. These considerations tend, it is true, to diminish our regret at the neglect which has been shewn by the medical community to Hunter's original writings, but they do not, nor can they excuse such neglect on the part of those who desire to be well informed. There is something both delighful and instructive in conversing with an original mind, even though we may be otherwise acquainted with the facts which *its* originality has developed.—The enthusiasm, the freshness, the boldness are conveyed in the manner as well as in the matter, and insensibly the noble infection is caught, and we long to tread in the footsteps of genius.

We hail, then, the publication of this new and excellent edition of all John Hunter's works, with feelings of the highest pleasure. At the same time that it has issued from the press, the new library and enlarged museum of the College of Surgeons, of London, have been opened, and a professor of comparative anatomy has been appointed, to display the scientific treasures which Hunter has accumulated. That museum, so displayed, is the noblest monument which can be erected to his memory, and surgery, within the walls of her college, witnesses the honours rendered by the profession to her favourite child.

We hope that the spirit exhibited in the college, will be equally exhibited out of it, and though all cannot have John Hunter's museum, all may possess his published works.

The first volume contains the Life of John Hunter, by Drewry Ottley—an Appendix to that Life, consisting of a Chronological History of Mr. Hunter's Writing's, of an Account of the Editions of Mr. Hunter's Works, and of Mr. Hunter's Evidence on the Trial of Capt. Donellan—and his Surgical Lectures, with Notes, by J. F. Palmer.

We extracted some of the more interesting anecdotes from his life in the last number of this Journal, and most of our contemporaries have extracted as liberally (or more so) as ourselves.

To the lectures on the principles of surgery, we shall allude more particularly in our next number, and therefore we say no more of them at present.

The Second Volume contains John Hunter's Treatise on the Natural History and Diseases of the Human Teeth, with Notes, by Thomas Bell—and the Treatise on the Venereal Disease, with Notes, by George S. Babington.

The illustrations to this Edition, comprehending 348 figures, in 61 plates, embrace the whole of those which accompanied the original publications, and, with the exception of those on the teeth, and two or three others, (which have been newly executed by Mr. Basire), are impressions from the original plates, which have been re-touched, by the permission of the Council of the Royal College of Surgeons, wherever it was found necessary, from the Hunterian drawings and preparations. To these have been added several others, together with the celebrated engraving of Hunter by Sharp, after Sir Joshua Reynolds, and a medallion head taken from the author's bust in the Royal College of Surgeons, by Flaxman.

The Fasciculus of Plates before us consists of the Portrait—Plates 2, 3, 5, 6, 7, 8, 10, 12, 13, 14, 15, 16, 18, 19, 21, 23, 52, 53, and 57, with corresponding explanatory letter-press.

It only remains for us to speak of the execution of this edition. It is highly creditable to all concerned. The editor, Mr. Palmer, has evinced great care, diligence, and judgment—the several annotators, selected for their knowledge in particular departments, have kept the promise which their names held out, and not kept it the less because they have refrained from burying the original text in annotations. On the whole, we cordially recommend all who have not an edition of Hunter's works, and who can possibly afford to buy a good one, to buy this. When the other volumes appear, we shall take care to notice them.

---

### NEW WORKS UPON ANATOMY AND PHYSIOLOGY.

I. THE CYCLOPÆDIA OF ANATOMY AND PHYSIOLOGY. Part X. June, 1836. Edited by ROBERT TODD, M.D., &c. &c.

This part has been long delayed, and the subscribers have evinced some degree of impatience at its non-appearance. It is the interest of publishers to ensure regularity in works which issue from the press in parts, for, if once distrust takes possession of the public mind, no such works will meet with subscribers.

The present part contains the following articles:—Electricity, Animal, continued, by Dr. Coldstream—Endosmose, by Dr. Dutrochet—Entozoa, by R. Owen, Esq.—Erectile Tissue, by Dr. Hart—Excretion, by Dr. Alison—Extremity, by Dr. Todd—Eye, by Dr. Jacob.

The most prominent articles are Entozoa, Excretion, and Eye.

Mr. Owen's account of the Entozoa is not unworthy of his scientific reputation. It is rich both in description and in illustrations, wood-cuts being profusely scattered through its columns.

Excretion, like all Dr. Alison's physiological essays, is a judicious compilation. The Doctor tells us all that is known in an agreeable manner, and, interspersed with the facts which his industry has collected, are sensible, and, not unfrequently, ingenious remarks.

Eye, by Dr. Jacob, is what might be expected from the discoverer of a membrane in that minute, but elaborate organ. The descriptions are accurate, the information brought down, or rather up, to a late level, the language intelligible and clear.

The part before us has not at all deteriorated. It is worthy of most of its predecessors, no trivial encomium. The work, when entire, will be amongst the most valuable of the day, and, even so far as it has gone, it should be possessed by every lover of anatomy.

II. Anatomie du Systeme Dentaire considerée dans l'Homme et les Animaux. Par Ph. Fr. Blandin, &c. &c. Bailliére, Paris.

This is a good work, and we shall notice it fully in connexion with other works upon the teeth, in our next number.

III. Nouvelles Recherches sur la Structure de la Peau. Par M. G. Breschet, &c. &c. Bailliére, Paris.

This work, also, will be analysed in our next. In the interim, we recommend it to our readers.

IV. Leçons d'Anatomie Comparée de Georges Cuvier. Seconde Edition. Crochard et Cie. Paris.

*Tome Premier contenant les Généralités et les Organes du Mouvement des Animaux Vertebrés.*

*Tome Quatriéme—Premiere Partie, contenant les Organes de Mastication, d'Insalivation et de Deglutition des Animaux Vertebrés.*

*Tome Quatriéme—Deuxieme Partie, contenant la Suite de l'Appareil de Chylification des Animaux Vertebrés.*

This second edition of the Leçons of Cuvier is now in process of publication, and we recommend all who are anxious to possess the lectures of that immortal man to purchase it. The information in it is brought to the existing level by his pupils. It is a great work and should be in every well-stocked library.

V. Anatomie Descriptive. Par J. Cruveilhier. Quatre Tomes. 8vo. Paris—Bechet Jeune.

Of all systems of human descriptive anatomy, this must be deemed not only the latest but the best. It is superior to Cloquet's, which, until it appeared, was the "codex," of late years, in this country. Why does not a translation of it appear? There will be one we are confident. The success of Knox's Cloquet, will be encouragement enough to any translator.

When we look on the preceding list, and it might be readily augmented, we see every reason for delight at the progress of scientific anatomy. The mode of studying, of describing, of viewing, and of teaching this science of facts, itself the basis of all science in medicine, has improved, is improving, and will, no doubt, continue to improve.

RECENT ANATOMICAL PLATES.

1. *Quain's Plates.*

These have lately been occupied with the Veins, and are now devoted to the Absorbents. They have greatly advanced in point of distinctness of execution since their commencement.—We would again press on their editor the necessity for making them *complete.* It will be wiser and better to publish twenty extra numbers, than to leave the "system" imperfect. When the work is finished, we anticipate a good sale for it. Purchasers now hang back from the dread of its not being eventually completed.

2. *The Plates of Bourgery.*

These are, in general, magnificent. But, unfortunately, the irregularity in their appearance and in their *mode* of publication is such, as materially to detract from their marketable value. One system is begun, abandoned before it is half completed, and another system is commenced, to be treated in the same manner.

Yet with all these drawbacks, the Plates of Bourgery will form an æra in anatomy, or, at all events, in anatomical engraving, and it could be wished that their price would put them within the reach of the majority of medical men. That is out of the question, for the work, when finished, will have cost between thirty and forty guineas.

---

RECENT WORKS ON DISEASES OF THE SKIN.

I. A PRACTICAL TREATISE ON DISEASES OF THE SKIN, &c. By SAMUEL PLUMBE, &c. Fourth Edition, Revised, Corrected, considerably Enlarged, and with Additional Engravings. 8vo. pp. 607. London, 1837.

II. A PRACTICAL COMPENDIUM OF THE DISEASES OF THE SKIN, &c. By JONATHAN GREEN, M.D.&c. Second Edition, Corrected and Improved, with Two Illustrative Plates.

Diseases of the skin cannot now be said to be neglected in this country nor in France. The lectures and the works of Alibert and of Rayer in the latter, the works of Willan and Bateman, the translation of Rayer, and the new editions of Plumbe's and of Green's books here, shew that the interest of the Profession and of the public has been fully awakened in reference to these disorders.

It is only a short time since the labours of the continental pathologists were neglected and undervalued in this country. Then we were constantly endeavouring to overcome the apathy, and to dispel the prejudices of the profession. Now we may remark something like the opposite extreme, and it is rather necessary to check than to encourage this tendency to look for all excellence abroad.

We are essentially a more practical people than our neighbours. The genius of our institutions has thrown men on their individual energies and exertions for success, and that habit of thought and action has been generated which looks always to positive and tangible results. On the Continent it has been otherwise. The more or less absolute character of the governments has based its policy on centralisation, and the repression of individual enterprise, and the national character has taken, both in politics and science, such a tone as might be expected from such a system. The visionary German and ingenious Frenchman display in a remarkable degree the want of that spirit of utilitarianism, which distinguishes the English character.

We would urge our readers not to imitate this disposition to abstraction and refinement, which the works of the continental pathologists present. We would urge them to form their tastes at home, and to look to our neighbours rather as an example of what an assiduous cultivation of pathology may effect, than as models of what physicians and surgeons should be. We have seen a good deal of those who have studied much abroad, and of those who are plunged over head and ears in foreign reading. With scarcely an exception, they have been ill adapted for success in practice in England. They have usually been more *au fait* at examining the dead than at curing the living.

Mr. Plumbe, we regret to add the late Mr. Plumbe, remarks in the Preface to the fourth edition of his book :

"The fact is unquestionable that our countryman Willan led the way in opening this part of the science to the world. His recorded facts and his published illustrations being of origin and date antecedent to that of the French school by several years. There have been numerous English contributors too, to the fund of information on these subjects ; and it is not a day of triumph to English practitioners when, in the study of these diseases, they are expected to peruse the works of continental authors in preference to those of their contrymen, under an unfounded assumption that they are more valuable and instructive than those of the latter.

There are two curious points of evidence affecting this question.

First, there has been for years, in a work published in the French capital, purporting to be largely occupied by descriptions of its public institutions, an uncontradicted statement, that though the hospital of St. Louis is dedicated to the reception and treatment of cutaneous diseases, furnished with baths and 'all appliances and means to boot,' yet the treatment adopted there is not found more successful than that adopted elsewhere under less favourable circumstances. Secondly, in that very institution, the treatment of a very large portion is surrendered by the physicians and surgeons into the hands of secret *nostrum-mongers*."

He observes too, and we think with some degree of justice, that :—

"There is commonly a wide difference between the delineations and descriptions of French and English authors on this class of diseases. As regards the delineations, either of Alibert or Rayer, if attentively examined, the utmost liberality must be exercised to enable us to admit them to be representations of individual diseases as occurring here and known by the same names, doubtless both these authors have strongly, if not faithfully, performed their tasks in this respect; but the delineations often appear to an Englishman to amount to caricatures, and the descriptions to abound with exaggerations.

That both may be correct, as regards the cases which come under the French practitioner's eye, it is far from my intention to deny, or even to doubt. It must be considered that the individual portraits are, for the most part, drawn from the persons of the lowest and poorest of the French population,—a class of people so poor and so badly fed in the main as would lead us, *à priori*, to expect a greatly aggravated form of those diseases among them. A slight examination of the extensive and clever illustrations of these authors will convince the reader of the correctness of my observations on this point; and really the descriptions are still more widely separated from the reality, as the diseases are seen in England, than the illustrations themselves."

Mr. Plumbe has made many additions to the work. The anatomy and physiology of the skin have been illustrated by the late researches of Breschet, two new figures have been added, and much has been quoted from Rayer. Altogether Mr. Plumbe's work, though not one of the first order in a scientific point of view, is, perhaps, better adapted for general perusal and for students, than the voluminous and prolix tome of Rayer.

Of Mr. Green's book it is not necessary to say much. The additions are not considerable—and the chief object is to urge the use of baths. They are highly valuable adjuncts to treatment, no doubt, and Dr. Green's sense of their utility is probably not diminished by his being a proprietor of them.

"I have been taken to task" says he, "by some of my reviewers for not entering more into detail relative to these remedial means ; suffice it to say, that in 1822, at the Hospital of St. Louis alone, at Paris, 127,752 of these baths were administered. In 1833, the number had increased to upwards of 150,000 in the year. In 1834 and 1835, upwards of 180,000 were administered each year.

In November, 1836, I was informed the numbers would be about the same, as the baths were always full; and of sixty-two patients whom I saw present themselves to the Baron Alibert, one morning, for consultation, only seven were prescribed for, exclusive of one or other of these baths as part of the treatment.

These numbers may surprise the reader, and the surprise will be increased when he considers that these baths are erected at the other hospitals, prisons, and poor-houses throughout Paris, where they are continually taken, not only as curative of disease, but as preservative of health.

In this country, it is true, no such opinion of the worth of vapour and fumigating baths at present exists among the members of the medical profession; yet I should imagine that the time was not far distant when their utility will be generally acknowledged; and in confirmation of this idea, I may say that I have lately superintended their erection at three of our metropolitan hospitals."

This is quite true, and the use of baths cannot be dispensed with by those who would attempt to treat eruptive disorders.

---

AN ADDRESS DELIVERED TO THE MEMBERS OF THE WORCESTERSHIRE NATURAL HISTORY SOCIETY, ON THE OPENING OF THE WORCESTERSHIRE MUSEUM, September 15, 1836. By CHARLES HASTINGS, M.D. &c. 8vo. Stitched. pp. 57.

Those who know Dr. Hastings best, would form the highest expectations of the eloquence, the enthusiasm, and the honourable feeling, of any address delivered by him to an association of scientific men. Such anticipations are amply realised in the Address before us. Its title so fully displays its objects that it is not necessary for us to insist on them, and all that we need do is to extract one or two passages which bear on points deserving of attention.

1. *Necessity of Scientific Associations in England.*

"There is also in the peculiar circumstances of England an additional motive for the formation of societies for the advancement of the study of nature. There is no civilized country in Europe of which the government has shewn so great an indifference with respect to the progress of science as that of Great Britain. Even in far less civilized and enlightened ages than the present, patronage was extended to science by the sovereigns of Europe; but it is a fact much to be regretted, that in this country, where, owing to our commercial and maritime affairs being on so extensive and vast a scale, its cultivation is of more importance than in any other European state, our Government has been supine on this subject, and little national encouragement has been given to those who have made important discoveries in it. Of all the countries of Europe, France is undoubtedly the one in which the scientific establishments have been conducted on the most enlightened and liberal principles, and in which science is most successfully cultivated. This high distinction she owes to the formation of the Institute at the close of the last century, and to which upwards of £100,000 is annually paid by the French Government. Owing to this circumstance many members of the Institute are in opulent circumstances, and can, without anxiety and in calm seclusion, pursue their scientific inquiries; or, if they desire to be distinguished in public life, all the honours of the State are open to philosophers and literary characters. The sage and the hero deliberate in the same cabinet; they are associated among the privy councillors of the king; they sit together in her house of peers and in her chamber of deputies; they bear the same titles, they are decorated with the same orders; and the arm and the mind of the nation are thus indissolubly united for its glory and for its defence."

What can be more eloquent, alas, what can be more true, than the preceding statements? Look at England and France in their respective encouragement of abstract science and philosophy, and blush for our own land. The evil is a crying one, and we hope that ere long it will work its remedy. This is a subject which deserves more than a paragraph, and which we shall dwell on more at large hereafter. The attention of the Legislature and of Government should be called to it.

2. *Labours and Objects of the Worcestershire Natural History Society.*

"It is now three years since the Worcestershire Natural History Society was instituted, and it has received during that period most encouraging support from all classes of the inhabitants. In order to secure the particular investigation of the several branches of the natural history of the county, it was determined at the commencement of the undertaking to divide the Society into sections; and in furtherance of these views, committees have been appointed each year on Statistics, Zoology, Botany, Geology, Mineralogy, and Meteorology. The labours of these committees have been very useful in collecting together valuable information, which otherwise would not have been brought forward."

The nucleus of a library is formed—a museum is commenced—some zeal has been kindled—and such addresses as this of Dr. Hastings will undoubtedly kindle more.

3. Dr. Hastings most ably points out the advantages likely to follow a more general acquaintance with natural history. He touches with a master's hand the lives of the great men who have travailed in its regions, and he particularly dwells on the works of the immortal Cuvier. All that we can extract is this slight tribute to the memory of that great man.

"The grave has now closed upon Cuvier, and his voice is no more heard among men, but he yet speaks through his writings, which will long continue to be regarded as striking specimens of what a devoted zeal for science, when directed by superior intellectual endowments, can effect in the advancement of useful knowledge. In every part of the civilized world the name of Cuvier is revered, and every where disciples of this great man are to be found who are animated by similar zeal, and who, by following up the investigations which he had begun, are continually making fresh additions to the Temple of Science."

---

An Examination of Phrenology; in Two Lectures, delivered to the Students of the Columbian College, District of Columbia, Feb. 1837. By Thomas Sewall, M.D. Professor of Anatomy and Physiology. 8vo. pp. 67. Washington, 1837.

Dr. Sewall is evidently a well informed, and, as evidently, a well intentioned man. He examines phrenology with no malice prepense, with no spirit of dogmatism, with no wish to bully. If he disputes the conclusions with the phrenologists, he does so after arguing the question with them, and the grounds of his dissent, as well as the process of reasoning which leads to it, are openly exposed. Yet withal these is some dry humour in the doctor. He observes for example:—

"The ground which phrenologists assume the right to occupy is so extensive, and the outlets for retreat are so numerous, that it is difficult to present an objection to the science, which cannot, upon the common principles of reasoning, be plausibly evaded. A few examples will illustrate the idea which I wish to convey.

If an individual has a large head, and his mental manifestations are unusually powerful, the case is brought forward as a proof of the truth of phrenology; but if the manifestations are feeble, it is said that the great size of the head is the result of disease, or that the brain is not well organized, or that other circumstances have exerted an influence in diminishing its power. If a small head is connected with a powerful intellect, it only proves that the brain, though small, is well organized, and acts with uncommon energy. If an individual has a particular propensity strongly marked in his character, and there is no corresponding development of the brain, it is said that the organ has not been thrown out by indulging its desires ; but if there is a large development of an organ, and no corresponding propensity, then it is contended that the germ of the propensity is there, but that it has been repressed by education, or other circumstances; or it is found that some counteracting organ is fully developed which neutralizes the first."

After remarking that temperament is thrown into the scale by the phrenologists, he proceeds :—

" When all these fail, in furnishing a satisfactory explanation, another method still more amusing is sometimes resorted to, in relieving phrenology from embarrassment. It may be illustrated by the following facts :

There is a celebrated divine now living, in Scotland, equally distinguished for his amiable disposition, his gigantic powers of mind, and the great moral influence which he exerts upon the Christian world. This individual, it is said, has the organ of destructiveness very largely developed, and not having any counteracting organ very large, it is contended by those who are acquaint- with the fact, that he manifests his inherent disposition to murder, by his mighty efforts to destroy vice and break down systems of error. In this way he gratifies his propensity to shed blood.

By a recent examination of the skull of the celebrated infidel Voltaire, it is found that he had the organ of veneration developed to a very extraordinary degree. For him it is urged, that his veneration for the Deity was so great, his sensibility upon the subject of devotion so exquisite, that he became shocked and disgusted with the irreverence of even the the most devout Christians, and that out of pure respect and veneration for the Deity, he attempted to exterminate the Christian religion from the earth."

He examines the doctrine in detail, and the conclusion to which he comes, is decidedly opposed to phrenology.—But the phrenologists are both able and willing to take up the cudgels for themselves.

